# Supplementary material for: Cross-species transcriptomic integration reveals a MIRO1-mediated macrophage–T cell axis in glioma
Source: Life Sci Alliance. 2026 May 13;9(8):e202603749. doi: 10.26508/lsa.202603749 (PMC13171295; doi:10.26508/lsa.202603749)
Supplement: Supplementary file 1 [file LSA-2026-03749_TableS1.docx]

| **Patient** | **Date** | **Sex** | **Age** | **Final Path** | **Sample** |
| --- | --- | --- | --- | --- | --- |
| P1 | 4/30/2024 | M | 50 | IDH-mutant grade 2 oligodendroglioma;1p/19q-codeleted | P1_Plain, P1_DMSO, P1_MR3 |
| P2 | 8/26/2024 | F | 29 | IDH-mutant grade 2 astrocytoma; P53 overexpressed; ATRX lost; GFAP positive | P2_DMSO,P2_MR3 |
| P3 | 8/27/2024 | F | 51 | IDH-wildtype grade 4 glioblastoma; ATRX intact; P53 overexpressed | P3_DMSO,P3_MR3 |

**Table S1. Patient and Sample Information.**
