# Supplementary material for: Cross-species transcriptomic integration reveals a MIRO1-mediated macrophage–T cell axis in glioma
Source: Life Sci Alliance. 2026 May 13;9(8):e202603749. doi: 10.26508/lsa.202603749 (PMC13171295; doi:10.26508/lsa.202603749)
Supplement: Reviewer comments [file LSA-2026-03749_review_history.pdf]

Life Science Alliance

Cross-Species Transcriptomic Integration Reveals a MIRO1-Mediated Macrophage-T Cell Axis in Glioma

Zehui Du, Menghan Li, Brandon H Bergsneider, Andy P. Tsai, Kwang Bog Cho, Lily H Kim, John Choi, Gordon Li, Tony Wyss-Coray, Michael Lim and Xinnan Wang

DOI: https://doi.org/10.26508/lsa.202603749

Corresponding author(s): Dr. Xinnan Wang (Stanford University)

Review Timeline:

Submission Date:	2026-04-27
Editorial Decision:	2026-04-29
Revision Received:	2026-04-29
Accepted:	2026-04-30

Scientific Editor: Tim Fessenden

Transaction Report:

(Note: With the exception of the correction of typographical or spelling errors that could be a source of ambiguity, letters and reports are not edited. The original formatting of letters and referee reports may not be reflected in this compilation.)

No full Peer Review Process File is available with this article, as the authors have chosen not to make the review process public in this case.

Please note that the manuscript was reviewed at *Review Commons* and these reports were taken into account in the decision-making process at *Life Science Alliance.*

April 29, 2026

RE: Life Science Alliance Manuscript #LSA-2026-03749

Dr. Xinnan Wang
Stanford University
Neurosurgery
1201 Welch Rd., MSLS Building A, Rm p105
Palo Alto, CA 94305

Dear Dr. Wang,

Thank you for submitting your revised manuscript entitled "Cross-Species Transcriptomic Integration Reveals a MIRO1-Mediated Macrophage-to-T Cell Signaling Axis Alginging with Immunosuppression in Glioma" to Life Science Alliance. As we noted in our offer conveyed by Review Commons, would be happy to publish your paper in Life Science Alliance pending revisions necessary to meet our formatting guidelines. Thank you for thinking of Life Science Alliance as a suitable venue for this intriguing work.

MANUSCRIPT ORGANIZATION AND FORMATTING:

To avoid unnecessary delays in the acceptance and publication of your paper, please read the following information carefully. Full guidelines are available on our Instructions for Authors page, https://www.life-science-alliance.org/authors

Submission of a paper that does not conform to Life Science Alliance guidelines will delay the acceptance of your manuscript.

-Please upload all figure files individually, including the supplementary figure files; all figure legends should only appear in the main manuscript file.
-Please add a Running Title in our system.
-Please upload your Table in editable .doc or Excel format.
-Please add a Summary Blurb/Alternate Abstract and a Category for your manuscript in our system.
-Please add the X and Bluesky handles of your host institute/organization, as well as your own, and/or one of the authors, in our system.
-Please add authors' contributions to our system also.
-Please upload a clean manuscript file without the track changes. The version that includes changes should be uploaded as a related manuscript file.
-Abstract should not exceed 175 words. Please correct accordingly and update the abstract in the system as well.
-Please consult our manuscript preparation guidelines https://www.life-science-alliance.org/manuscript-prep and make sure your manuscript sections are in the correct order.
-Please rename "Declaration of Interests" to "Conflict of Interest."
-Please add your main, supplementary figure, and table legends to the main manuscript text after the references section.
-Please include the GEO accession numbers for raw sequencing data in the Data Availability section, as mentioned in your cover letter.
-LSA does not permit citation of "data not shown," "manuscript in preparation," "manuscript submitted," etc., in any section of the manuscript. Please include a citation for Sainz et al (such as a preprint), or reword the claims on line 65 to omit this reference.

We encourage our authors to provide original source data, particularly uncropped/-processed electrophoretic blots and spreadsheets for the main figures of the manuscript. If you would like to add source data, we would welcome one PDF/Excel-file per figure for this information. These files will be linked as supplementary "Source Data" files.

It is Life Science Alliance policy that if requested, original data images must be made available to the editors. Failure to provide original images upon request will result in unavoidable delays in publication. Please ensure that you have access to all original data images prior to final submission.

If you are planning a press release on your work, please inform us immediately to allow informing our production team and scheduling a release date.

We welcome submissions of potential cover images for the issue of LSA in which your work would appear. If you have high quality images associated with this work, please feel free to email these, with a caption, to the journal office.

LSA encourages authors to provide a 30-60 second video where the study is briefly explained. These videos will be appear embedded with the manuscript online at Life Science Alliance, and on social media to promote the published paper and the presenting author (for examples, see https://docs.google.com/document/d/1-UWCfbE4pGcDdcgzcmiuJl2XMBJnxKYeqRvLLrLSo8s/edit?usp=sharing). Corresponding or first-authors are welcome to submit the video. Please submit only one video per manuscript. The video can be emailed to contact@life-science-alliance.org

FINAL FILES:

To upload the final version of your manuscript, please log in to your account: https://lsa.msubmit.net/cgi-bin/main.plex
You will be guided to complete the submission of your revised manuscript and to fill in all necessary information. Please get in touch in case you do not know or remember your login name.

The following items are required for acceptance.

-- An editable version of the final text (.DOC or .DOCX) is needed for copyediting (no PDFs).

-- High-resolution figure, supplementary figure and video files uploaded as individual files: See our detailed guidelines for preparing your production-ready images, https://www.life-science-alliance.org/authors

-- Summary blurb (enter in submission system): A short text summarizing in a single sentence the study (max. 200 characters including spaces). This text is used in conjunction with the titles of papers, hence should be informative and complementary to the title. It should describe the context and significance of the findings for a general readership; it should be written in the present tense and refer to the work in the third person. Author names should not be mentioned.

The license to publish form must be signed before your manuscript can be sent to production. A link to the license to publish form will be available to the corresponding author only. Please take a moment to check your funder requirements.

Reviews, decision letters, and point-by-point responses associated with peer-review at Life Science Alliance will be published, alongside the manuscript. If you do want to opt out of having the reviewer reports and your point-by-point responses displayed, please let us know immediately.

Thank you for your attention to these final processing requirements. Please revise and format the manuscript and upload materials as soon as you are able.

Thank you for this interesting contribution to the literature. We look forward to publishing your paper in Life Science Alliance.

Sincerely,

Tim Fessenden
Scientific Editor
Life Science Alliance

April 30, 2026

RE: Life Science Alliance Manuscript #LSA-2026-03749R

Dr. Xinnan Wang
Stanford University
Neurosurgery
1201 Welch Rd., MSLS Building A, Rm p105
Palo Alto, CA 94305

Dear Dr. Wang,

Thank you for submitting your Research Article entitled "Cross-Species Transcriptomic Integration Reveals a MIRO1-Mediated Macrophage-T Cell Axis in Glioma". It is a pleasure to let you know that your manuscript is now accepted for publication in Life Science Alliance. Congratulations on this interesting work and thank you for working with us so quickly to ready your paper for publication.

The final published version of your manuscript will be deposited by us to PubMed Central upon publication.

Your manuscript will now progress through copyediting and proofing. It is journal policy that authors provide original data upon request.

Your article will publish open access upon publication under a CC-BY license.

I understand you have posted the reviewer reports and your point-by-point responses to the related preprint of this work on BioRxiv, and that LSA will not publish these alongside your paper.

IMPORTANT: If you will be unreachable at any time, please provide us with the email address of an alternate author. Failure to respond to routine queries may lead to unavoidable delays in publication.

Scheduling details will be available from our production department. You will receive proofs shortly before the publication date. Only essential corrections can be made at the proof stage so if there are any minor final changes you wish to make to the manuscript, please let the journal office know now.

DISTRIBUTION OF MATERIALS:
Authors are required to distribute freely any materials used in experiments published in Life Science Alliance. Authors are encouraged to deposit materials used in their studies to the appropriate repositories for distribution to researchers.

You can contact the journal office with any questions, contact@life-science-alliance.org

Again, congratulations on a very nice paper. I hope you are pleased with how the manuscript was handled editorially. We look forward to future exciting submissions from your lab.

Sincerely,

Tim Fessenden
Scientific Editor
Life Science Alliance
